# Construction and evaluation of China older-adult care service smart supply chain system

**DOI:** 10.3389/fpubh.2023.1249155

**Published:** 2023-11-24

**Authors:** You-Yu Dai, Guanlong Liu, Long Sun

**Affiliations:** International Business School of Shandong Jiaotong University, Weihai, China

**Keywords:** older-adult care services, old-age security, smart supply chain, service demand, China

## Abstract

**Introduction:**

As the aging of the population continues to deepen, the pressure on social pensions is gradually increasing, and the issue of assistance has become a problem that must be solved. With the development of science and technology, people’s living standard is constantly improving. The older-adult care services expected by the older-adult are wider than meeting the basic daily needs of individuals. The current industry should also consider combining modern science and technology with the older-adult care service industry to serve older-adult better and enable older-adult care service providers to move towards the service needs that make people happier and healthier. This research is about constructing and evaluating China’s older-adult care services smart supply chain.

**Methods:**

Based on the research results of previous scholars, this paper divides the Sun construction of the smart supply chain of China endowment service into four aspects: policy aspect, economic aspect, social aspect, and technical aspect; the four significant elements are divided into the first-level indicators, and 16 second-level indicators are divided under the first-level indicators. The importance and satisfaction of each evaluation index were obtained by distributing questionnaires to the managers who study the supply chain and the employees who are related to the old-age service.

**Results and discussion:**

After the reliability analysis, the importance-performance analysis (IPA) quadrant analysis chart of the evaluation index was constructed using importance-performance analysis. The index of creating a smart supply chain system for China’s old-age service is given priority, the supply chain system of China’s old-age care service is further improved, and the social security of China’s old-age service is enlarged.

## 1 Introduction

With the continuous development of the social economy and technology, various fields have achieved unprecedented improvements in China (1). However, with the constant deepening of population aging, the pressure on social older-adult care is also increasing year by year, and the issue of older-adult care has become a problem that needs to be solved in China (2, 3). According to the latest data from the Chinese 7th National Population Census, as of November 1, 2020, the total population in China was 1411.78 million, including 264.02 million people aged 60 and above, accounting for 18.70% of the total population. Compared with the 6th National Population Census data in 2010, the increase was 5.44 percentage points (4). The implementation of the national strategy of active population aging is related to the overall development of the country, the well-being of billions of people, and has significant and far-reaching significance for the sustained and healthy development of Chinese economy and society during the 14th Five Year Plan period and beyond. We should respond to population aging with a more positive attitude, policies, and actions (5).

The issue of population aging is not only severe in China but also in the whole world. However, traditional older-adult care models can no longer meet the current needs of older-adult care (2). Traditional older-adult care services include home, institutional, and community care. With the high cost and low quality of older-adult care services becoming increasingly prominent, the existing operational models of older-adult care services can no longer meet the diverse needs of the aging population (2, 6). In terms of the current situation of older-adult care, older-adults have a higher level of pursuit for realizing their own life value and cultural, entertainment, and spiritual aspects (7, 8), not only limited to meeting basic living needs such as catering and medical care in the past.

Based on the continuous popularization of information digitization, utilizing Internet technologies such as big data, cloud platforms, and the Internet of Things to achieve service combinations has become an effective way to innovate innovative older-adult care service models (9, 10). At present, there is relatively little research on innovative older-adult care services supply chain in the past 5 years (11–13). Therefore, in academia and industry, efforts are being made to study the construction of an smart supply chain system for older-adult care services (14–16).

The British Life Trust was the first to propose the concept of “smart retirement.” Demiris and Hensel (17) pointed out that thoughtful home care is a service model that uses intelligent service equipment and information technology as carriers to systematically provide remote monitoring services for older people to enhance their self-care ability and older-adult care level. Lemlouma et al. (18) constructed a dependent intelligent older-adult care system framework and then analyzed the particular service provision projects and times for each older-adult based on scientific evaluation, providing different services according to their situations. Chan et al. (19) believed that innovative older-adult care services should integrate multiple social resources to build an Internet home care service system centered on older persons, which includes a unified collection of related technologies, services, and information integration.

Zuo and Chen (20) conducted a survey and research on hundreds of older people in society, dividing home-smart older-adult care into five different dimensions: daily care, economic support, safety protection, rehabilitation and health care, and spiritual comfort. Then, they constructed a high-quality, high-efficiency, and low-cost intelligent older-adult care service to meet the development needs of an aging society. Li et al. (21) believed that a new smart older-adult care service system should be constructed suitable for the current social development in China, and the critical role played by traditional older-adult care models cannot be ignored. He advocates optimizing the allocation of various social resources, encouraging older people to strengthen self-learning, improve their acceptance, and meet older people’s older-adult care service needs.

Meng et al. (22) conducted a study on the practicality of social networking sites browsed by older-adults and found that the sites frequently browsed by older people currently have shortcomings. Therefore, it is necessary to continuously transform and upgrade these social networking sites to make their service functions healthier and more informative, enabling older persons to receive practical guidance and assistance when browsing social networking sites. Making social networking sites for older people more functional and scientific and better meet older-adults’ convenient use of social networking sites. Zeng and Hou (23) found that Chinese policies on innovative older-adult care services are immature. The incentive policies for older-adult care services need to be strengthened, and the review and supervision policies for the smart older-adult care service system need to be improved to ensure the sustainable and healthy development of innovative older-adult care.

Chen (24) built a competent older-adult care comprehensive service platform and designed a conceptual model of the intelligent older-adult care supply chain to provide constructive suggestions for effectively integrating various elements such as older-adult care service node enterprises, older-adult care logistics, older-adult care fund flow, older-adult care information flow, etc., guiding and stimulating older-adult care demand, monitoring and improving older-adult care supply, and ensuring the realization of the value co-creation and value appreciation of older-adult care services. On this basis, design five mechanisms: integration mechanism, guidance mechanism, coordination mechanism, cooperation mechanism, and incentive mechanism to ensure the healthy, stable, and sustainable operation of the intelligent older-adult care service supply chain.

Dong (25) mentioned that with the development of older-adult care socialization and the increasing demand for older-adult care services, how to provide affordable older-adult care services has become a problem that the government and various fields of society must consider. Therefore, it is necessary to integrate resources from all parties reasonably, build an excellent older-adult care service supply chain that meets the needs of older persons, meet their older-adult care needs, improve their quality of life, and achieve the rational allocation of resources and the integrated development of the older-adult care machine logistics industry to avoid resource waste.

According to the construction of innovative older-adult care service supply chains by many scholars, they can be divided into four aspects: factors from policies, elements from the economy, factors from society, and factors from technology. Among them, the policy factors include Che and Zhou’s (26) improving the incentive mechanism, standardizing process management, and conforming to the evaluation and supervision criteria. Chen (24) proposed that enterprise qualifications should be complete. Economic factors include Ma et al.’s (27) ensuring service profits. Liu (28) proposed that the quality of employees should be high. Dong (25) proposed that product quality is guaranteed. Social factors include Wang and Chen (29) indicated that the base of older-adult care entities is large. Shen (30) proposed that the current development situation should be rapid. Chen (24) proposed that there should be more social older-adult care enterprises. Shen (30) proposed that the target audience should have a higher level of acceptance. Technological factors include Zhu’s (31) proposal to promote the popularization of the Internet of Things. Demiris and Hensel (17) proposed the deepening of artificial intelligence. Song presented in 2022 that cloud computing responds quickly. Zeng and Hou (23) proposed that the scale of extensive data collection is large. Meng et al. (22) proposed that mobile communication has broad coverage.

Based on the issues and current situation of the older-adult care service smart supply chain proposed by other scholars, this study defines the “older-adult care service smart supply chain” as: utilizing advanced technologies and equipment such as the Internet of Things, the Internet, and intelligent devices, guided by the diverse and personalized needs of older-adult care service demanders. In order to coordinate the interests of all parties and bridge the supply–demand imbalance of older-adult care services, it utilizes logistics, capital flow, information flow, and service flow in the supply process of older-adult care services, organically connecting various functional older-adult care service providers, older-adult care service integrators, and older-adult care service demanders. This older-adult care service smart supply chain would provide real-time, fast, efficient, and low-cost older-adult care service network structure for older-adult care service demanders. This article summarizes that the China older-adult care service smart supply chain is divided into four parts, as shown in Table 1.

**Table 1:** Establish an indicator system.

Factors from policies	Improvement of incentive mechanism	(26)
	Completeness of enterprise qualifications	(24)
	Process management should be standardized.	(26)
	The evaluation and supervision criteria should be consistent	(26)
Factors from economy	Guaranteed service profit	(27)
	The quality of employees should be high	(28)
	Product quality guaranteed	(25)
Factors from society	Large base of older-adult care entities	(29)
	The current development situation needs to be rapid	(30)
	More social older-adult care enterprises	(24)
	The acceptance level of the target audience	(21)
Factors from technology	The popularization of the Internet of Things	(31)
	Deepening of artificial intelligence	(17)
	Cloud computing responds quickly.	(32)
	The large scale of extensive data collection	(23)
	Broad coverage of mobile communication	(22)

Our current primary task is to effectively integrate modern information technology into the older-adult care service industry and e-commerce industry (33), create a smart supply chain system for older-adult care services (34), and apply it to older-adult care services in China to alleviate the aging problem in China effectively (16, 35, 36). Through continuous innovation and in-depth study on the smart supply chain of older-adult care services in China, we can effectively solve older-adult care problems and promote the social older-adult care security theory in China, which has particular significance (37, 38). The older-adult population is different from other groups and belongs to a relatively vulnerable group in society. Therefore, establishing a comprehensive China older-adult care service supply chain system can effectively improve the older-adult care service capacity in China (2).

By surveying staff serving the older-adult population in China, the supply chain system for older-adult care services in China can be further improved, thereby increasing the social security for older-adult care in China (3, 39, 40). The older-adult population and staff serving older-adults should provide objective feedback on various information and indicators to make more sweeping changes and innovations to the smart supply chain system of older-adult care services based on the feedback content and improve the living standards of older persons (23).

## 2 Research design

The research aim of this article is to construct and evaluate the smart supply chain for older-adult care services in China. The research tries to analyze the smart supply chain system for older-adult care services in China based on its four significant aspects of construction and propose reasonable suggestions to optimize the smart supply chain system for older-adult care services in China and alleviate the burden of older-adult care in China. This article mainly divides the smart supply chain system for older-adult care services in China into four major parts, which are from four aspects: policy, economy, society, and technology. At the same time, the second level title is extended according to these four aspects. For example, policy elements include improvement of incentive mechanisms, integrity of enterprise qualification, standardization of process management, and consistency of evaluation and supervision criteria.

According to Dai et al. (41) and Zhang et al. (42), the Delphi method and IPA method are taken as the theoretical basis. The Delphi method is often used to clarify the outline of indicator systems (43). Many previous studies adopted IPA to evaluate their index systems. Such as in the academic field of education (44), internal public space quality in affordable housing (45), tourism (46), consumption (47), supply chain (48), and so on.

### 2.1 Delphi method

The primary indicators in this article include four indicators: factors from policies, aspects from the economy, factors from society, and factors from technology (30). Based on the four primary indicators, 16 are differentiated, and the secondary indicators correspond to the primary ones. Firstly, a preliminary survey questionnaire was designed and distributed to experts related to the research on the smart supply chain system for older-adult care services in China. Their processing results were obtained. Based on their processing results, the 80% pass rate indicator was retained, and indicators below 80% were modified until the pass rate exceeded 80% (49).

The selection methods for consulting experts in this article mainly include (1) practitioners engaged in China’s older-adult care services, (2) social research scholars engaged in researching the smart supply chain of China’s older-adult care services, and (3) school teachers engaged in researching the supply chain. Twelve experts were selected for the consultation questionnaire.

### 2.2 Questionnaire design and sampling

The questionnaire design in this article has two dimensions, one is importance, and the other is performance. Each index has 20 questions, totaling 40 questions. According to Zhao et al. (45), Likert’s 5-Point scale assessed the importance and performance of building an smart supply chain system for older-adult care services in China.

A survey questionnaire is a way to help obtain raw data, mainly expressed through standardized data and designed questioning methods, to achieve the purpose of the survey. This questionnaire is intended to construct an smart supply chain system for older-adult care services in China, considering multiple factors such as research background and survey subjects and combining them with one’s research objectives. The survey in this study adopts a nonrandom sampling method, which is a judgmental sampling in nonrandom sampling. A questionnaire survey is distributed to individuals with specific experience or knowledge. As the research focuses on the construction and evaluation of the smart supply chain system for older-adult care services in China, to ensure the authenticity, reliability, and authority of the research results, the scope of the research object is determined to include relevant personnel studying the smart supply chain system for older-adult care services in China and workers participating in older-adult care services in China. Survey questionnaires were obtained through the online survey platform “Wenjuanxing.”

After the expert group discussed the survey questionnaire, it was believed that the overall content of the questionnaire design could build an smart supply chain system for older-adult care services in China, and the questionnaire was only distributed. The survey questionnaire was broadcast on November 29, 2022, and 198 questionnaires were collected. All valid questionnaires were sorted and organized, and data entry and statistical analysis were conducted using the statistical software SPSS 24.0. The questionnaire was divided into two parts:

The first part is to investigate the problems related to each variable in the construction of the China older-adult care service smart supply chain system and put forward the relevant questions about the importance and performance of each variable to obtain the significance and expressiveness of each variable. The second part of the content should mainly investigate the questionnaire participants’ information, such as their age distribution, occupation, and years of work related to older-adult care services.

### 2.3 IPA statistical methods

Importance and Performance Analysis (IPA) is the analysis of participants’ perception of the importance and performance of a particular process to find ways to improve their satisfaction and loyalty (50). The primary purpose of the importance-performance analysis method is to provide the characteristics of the enterprise’s product or service quality, which can provide opportunities for continuous improvement. Therefore, participants must evaluate the importance and expressiveness of the product or service quality characteristics through questionnaires.

## 3 Research results

### 3.1 Descriptive statistical analysis

This survey questionnaire was officially conducted, and 198 copies were distributed. The specific population statistics are shown in Table 2. The result is in line with Zhong and Wang (51) and Wu (52). Such precious studies all conclude that talents in older-adult service industry are lack, low-education level, and significant differences in age structure. So the result questionnaire is representative.

**Table 2:** Demographics.

Variable	Items	Number of people	Percentage (%)
Gender	Male	88	44.44
	Female	110	55.56
Age	Aged 21-30-year-old	70	35.35
	Aged 31-40-year-old	54	27.27
	Aged 41-50-year-old	24	12.12
	Aged 51-60-year-old	26	13.13
	Aged more than 60 years old	24	12.12
Educational level	College/Junior College	106	53.54
	Master’s degree	60	30.3
	Doctor’s degree	32	16.16
Occupation	School Teacher	70	35.35
	Enterprise staff	84	42.42
	government agent	44	22.22
	manager	60	30.3
	Front-line staff	94	47.47
	Government staff	44	22.22
Working experience	Less than 1 year	62	31.31
	1–5 years	60	30.3
	More than 5 years	76	38.38

### 3.2 Reliability and validity analysis

#### 3.2.1 Reliability test

In this pre-survey, the importance and performance are divided into four levels: from the policy aspect, from the economic part, from the social aspect, and from the technical feature. Reliability analysis mainly evaluates the reliability of the overall questionnaire. The results of this study show that in terms of importance, the Cronbach’s α value is 0.949, while the value of performance is 0.987. The α value must be at least greater than 0.5, and α value more excellent than 0.7 indicates high reliability. The research results show that each level meets high-reliability criteria, so the measurement reliability of the importance and performance of this questionnaire is good. The test results are detailed in Table 3.

**Table 3:** Cronbach’s alpha scale pre-survey.

Layer Name	Importance Cronbach’s α	Performance Cronbach’s α
From policy perspective	0.847	0.963
From economic perspective	0.872	0.938
From social perspective	0.860	0.946
From technical perspective	0.879	0.959

#### 3.2.2 Validity test

Expert validity: Determine the final indicator by combining relevant research from previous scholars and scoring experts based on questionnaires. Content validity: Based on an extensive review of prior literature, various factors are summarized to form a survey questionnaire. Experts and scholars then graded and tested whether the questionnaire design conforms to the research topic. Experts and scholars assisted in the clarity, face validity, and content validity of the questions in this scale, which helped to make the seal more accurate. Therefore, 12 participants conducted a content relevance assessment on constructing an smart supply chain system for older-adult care services in China, evaluated the suitability between each cross-sectional area and the measurement questions, and checked whether the questions represented the module. The items were added or reduced through expert discussions, merged or differentiated, and evaluated and revised. The initial questionnaire was formed. Then distribute the questionnaire. The questionnaire results have good validity.

### 3.3 IPA of the construction of China older-adult care service smart supply chain system

This paper uses IPA analysis to explore the expressiveness evaluation of each indicator for the construction of the China older-adult care service smart supply chain system by taking the average of importance and performance as the dividing point, cutting the space into four quadrants on the X and Y axes, taking importance as the X axis and performance as the Y axis.

**Figure 1:**
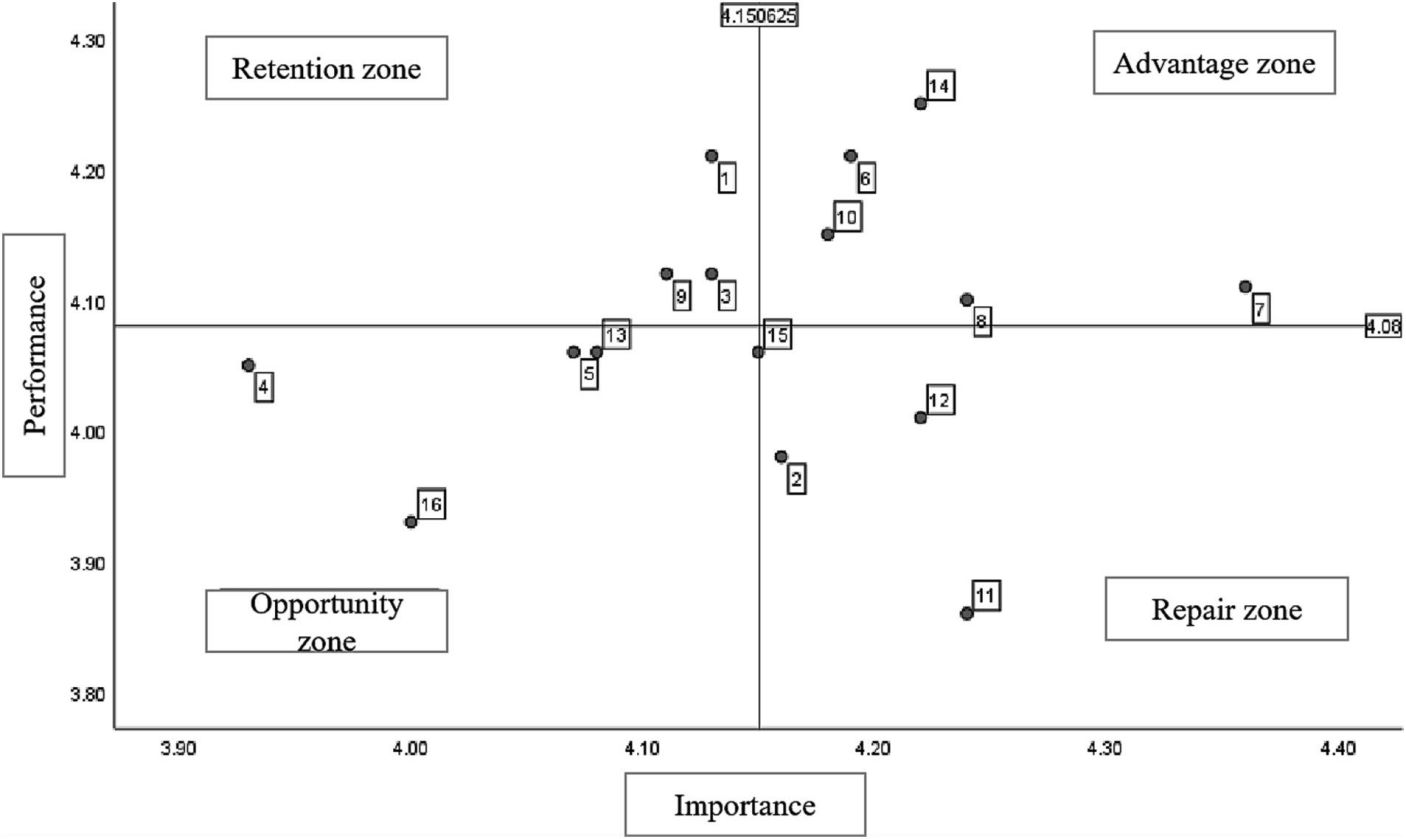
IPA analysis diagram of this research.

To present the importance and performance of each indicator in constructing the China older-adult care service smart supply chain system, this study revised the IPA analysis method studied by Chen (53), so the overall average value of the importance and performance of each indicator is taken as the standard to distinguish the four quadrants so that the extent and performance of each hand can be more intuitively compared. Therefore, the overall average value of 4.06 of importance is taken as the X coordinate, and the overall average value of 4.15 of performance is taken as the Y coordinate as the central intersection point. A scatter diagram of four quadrants is made accordingly. See Table 4 and Figure 1 below for specific values.

**Table 4:** The coordinate values of each index.

Level	Indicators	Importance X		Performance Y		Quadrant
		Mean	Standard deviation	Mean	Standard deviation	
Policy	1. Improvement of incentive mechanism	4.13	0.66	4.21	0.67	Second (−, +)
	2. Completeness of enterprise qualifications	4.16	0.68	3.98	0.62	Fourth (+, −)
	3. process management should be standardized	4.13	0.72	4.12	0.65	Second (−, +)
	4. The evaluation and supervision criteria should be consistent	3.93	0.63	4.05	0.73	Third (−, −)
Economy	5. Guaranteed service profit	4.07	0.66	4.06	0.71	Third (−, −)
	6. The quality of employees should be high	4.19	0.68	4.21	0.65	First (+, +)
	7. Product quality guaranteed	4.36	0.73	4.11	0.63	First (+, +)
Society	8. Large base of older-adult care entities	4.24	0.72	4.10	0.64	First (+, +)
	9. Rapid development status quo	4.11	0.66	4.12	0.65	Second (−, +)
	10. More social older-adult care enterprises	4.18	0.61	4.15	0.63	First (+, +)
	11. Acceptance level of the target audience	4.24	0.62	3.86	0.65	Fourth (+, −)
Technology	12. Popularization of the Internet of Things	4.22	0.64	4.01	0.75	Fourth (+, −)
	13. Deepening of Artificial Intelligence	4.08	0.73	4.06	0.71	Three (−, −)
	14. Cloud computing responds quickly	4.22	0.64	4.25	0.61	First (+, +)
	15. Large-scale extensive data collection	4.15	0.61	4.06	0.62	Third (−, −)
	16. Wide coverage of mobile communication	4.00	0.62	3.93	0.72	Third (−, −)

The IPA analysis chart can be divided into four quadrants. The first quadrant is the “advantage zone,” indicating that constructing an smart supply chain system for older-adult care services in China is essential, and the results are satisfactory. Therefore, as long as the existing services are maintained, it is sufficient. The second quadrant is the “maintenance zone,” indicating that the construction of an smart supply chain system for older-adult care services in China only needs to continue to maintain this element without investing excessive resources to avoid resource waste. The third quadrant is the “opportunity zone,” the improvement factors in this quadrant are a secondary focus, indicating that this element is not very important and does not need to be urgently addressed. The fourth quadrant is the “repair area,” meaning that relevant personnel believe this aspect is essential in building an smart supply chain system for older-adult care services in China. Still, there has been no improvement, so priority must be given to improvement and strengthening.

## 4 Discussion

According to the analysis of the secondary evaluation indicators, in terms of policy, the importance of improving incentive qualification and process management in the smart supply chain system of China’s older-adult care service is not high. However, the performance is relatively high, which indicates that the current situation of China’s older-adult care is more suitable and only needs to continue to be maintained and improved. This result is consistent with Fang et al. (2). The consistent importance and performance of the criteria for evaluating and supervising innovative older-adult care services are not high. The above indicates that its proportion in the supply chain of innovative Chinese older-adult care services is unimportant and can be postponed. The importance of improving the qualifications of older-adult care enterprises is relatively high. However, the performance is low, indicating that enterprises’ capability is still essential in innovative older-adult care services. The qualification of older-adult care enterprises is relatively neglected and needs attention. This result echoes to Rinkinen (54).

In terms of economy, the importance and performance of high-quality employees and guaranteed quality of pension products are relatively high, which indicates that in the smart supply chain of Chinese innovative pension services, the quality of pension employees and products is currently in line with the status quo of pension in China. The results are consistent with Bao et al. (55) and satisfactory so that they can be maintained. The importance and performance of the service profit guarantee of the older-adult care industry are relatively low. This paper believes that, at present, the intelligent older-adult care service industry in China is in the initial stage of development, and the service profit obtained by it is at a low setting. It is still necessary to guarantee the most basic older-adult care service profit.

In terms of society, the large base of older-adult care subjects and the high importance and performance of the social older-adult care industry indicate that the current China older-adult care service smart supply chain system is still perfect for such a large number of older-adult care groups. The number of older-adult care enterprises in society is also increasing (6). To ease the burden of older-adult care, it needs to continue to be maintained. The rapid development of China’s intelligent older-adult care is of low importance but high performance, which indicates that the product of China’s innovative older-adult care service supply chain is in a relatively rapid stage of development (16). The importance of the acceptance of target objects is relatively high. Still, the performance could be higher, which indicates that the acceptance of target objects to intelligent pensions is low. As previous studies pointed out, China should increase the publicity of intelligent pensions and the acceptance of target objects to smart pensions [e.g., (56, 57)].

In terms of technology, the importance and performance of the rapid response of cloud computing are relatively high, which indicates that the cloud computing of the Chinese intelligent older-adult care service supply chain is relatively fast and can perfectly solve the analysis of various data of China competent older-adult care. This is the same as the expression of Griebel et al. (58). Unlike the trend of environmental change, the deepening of artificial intelligence, the comprehensive coverage of mobile communication, and the large scale of extensive data collection are unimportant. However, the performance is very high, which indicates that the three aspects of the China older-adult care service smart supply chain system perform well and can well solve the current older-adult care pressure in China. The importance of the popularization of the Internet of Things is very high. However, its performance is low, which indicates that although the Internet of Things is used very widely in the supply chain (59), it is not very popular in the smart supply chain system of China’s older-adult care services. Therefore, the popularization of the Internet of Things needs to be strengthened to facilitate the life of the older-adult care groups in China.

## 5 Conclusion and suggestions

### 5.1 Research conclusion

This article analyzes and summarizes the four aspects of building a smart supply chain system for older-adult care services in China, namely from the policy aspect, from the economic part, from the social aspect, and from the technical aspect. We identify the advantages and disadvantages of these four aspects to optimize the Chinese smart older-adult care service system in the future. The main conclusion is to enhance the target audience’s acceptance of intelligent older-adult care. Only in this way can the China older-adult care service smart supply chain system develop more healthily. At the same time, it is necessary to strengthen the qualification review of older-adult care enterprises to prevent damaging enterprises from affecting the smart supply chain system of older-adult care services in China. For the Internet of Things, it is also necessary to make it more universal to ensure real-time supervision of older people’s health, life, and well-being.

### 5.2 Research implications

At present, the smart supply chain system for older-adult care services in China is in the initial stage of development and has excellent potential for growth in the future. In recent years, the country has attached increasing importance to older-adult care, which is not only a problem in China, but also a problem that needs to be solved nationwide and globally. By building an smart supply chain system for older-adult care services in China, it can effectively alleviate the pressure on older-adult care, better allocate resources, and rationalize their application. At the same time, this is also highly beneficial for the older-adult population, making their lives more convenient and allowing them to feel more happiness.

From the IPA analysis chart, it can be seen that we need to leverage our advantages to provide sufficient and high-quality older-adult care service products and personnel from the supply side and make good use of cloud computing. We must maintain industry incentive mechanisms and standardized management processes to respond to the rapidly growing market. Through the research on the older-adult care service smart supply chain system, it was found that we need to leverage our advantages to provide sufficient and high-quality older-adult care service products and personnel from the supply side and make good use of cloud computing. Regarding the system, it effectively combines the two themes of “smart” and “older-adult care” and synergizes the functional advantages of these two aspects. This not only conforms to the trend of the times and the current total coverage model of e-commerce but also is conducive to promoting the development of an smart supply chain in China, making the fields covered by general interest more diversified, increasing the integration of public welfare into the market, and continuously deepening the degree of benefiting the people. We must maintain industry incentive mechanisms and standardized management processes to respond to the rapidly growing market. For the opportunities in the older-adult care service market, the industry needs to grasp its pulse, establish sound evaluation criteria, and ensure that the service profits of the industry are guaranteed. The technological development of artificial intelligence, big data, and mobile communication should be utilized more up-to-date. Practitioners should improve their professional qualifications and promote the new transformation of older-adult care services to target groups by popularizing the Internet of Things.

Intelligent older-adult care supply should not only comply with the development trend of the times but also meet the practical needs of the older-adult population, Optimize and adjust the relationship between supply and demand. In terms of building intelligent older-adult care teams, a team management mechanism that integrates responsibility, power, and benefits should be established. Customers attach great importance to the service provided in the supply chain. In this regard, members should be encouraged to fight, show correct service concepts, and have a long-term perspective so that companies can share profits and risks in the intelligent older-adult care supply chain with a shared future.

### 5.3 Research limitations and future recommendations

Although this study has achieved some results, there are still certain research limitations. Firstly, in terms of methodology, this study only used IPA analysis as an analytical method and did not introduce other analytical methods, which may also lead to slight errors in indicator analysis (60). Secondly, this study is only based on a small portion of research data, which has significant limitations for China Province. Therefore, this paper suggests that future research should combine various research methods to explore the importance and performance of indicators to obtain more accurate data. At the same time, statistical analysis should be conducted on the data from China to enhance its persuasiveness.

## Data availability statement

The original contributions presented in the study are included in the article/supplementary material, further inquiries can be directed to the corresponding author.

## Author contributions

Y-YD: conceptualization, methodology, validation, formal analysis, resources, data curation, and writing—review and editing. GL: investigation. Y-YD and GL: writing—original draft preparation. LS: supervision. All authors contributed to the article and approved the submitted version.
